# Association of apolipoproteins A1 and B with type 2 diabetes and fasting blood glucose: a cross-sectional study

**DOI:** 10.1186/s12902-021-00726-5

**Published:** 2021-04-01

**Authors:** Liang Gao, Yaju Zhang, Xingmin Wang, Hongli Dong

**Affiliations:** 1 Department of Clinical Laboratory, Affiliated Maternity & Child Health Care Hospital of Nantong University, Nantong, 226018 Jiangsu Province China; 2 Finance Section, Affiliated Traditional Chinese Medicine Hospital of Nantong University, Nantong, 226018 Jiangsu Province China; 3 Nantong Institute of Genetics and Reproductive Medicine, Affiliated Maternity & Child Health Care Hospital of Nantong University, Nantong, 226018 Jiangsu Province China; 4 Scientific Education Section, Affiliated Maternity & Child Health Care Hospital of Nantong University, Nantong, 226018 Jiangsu Province China

**Keywords:** Apolipoprotein A1, Apolipoprotein B, Apolipoprotein B/A1 ratio, Type 2 diabetes, Fasting blood glucose

## Abstract

**Background:**

Apolipoprotein (Apo) may be associated with type 2 diabetes (T2D), however, little is known whether or not serum apolipoproteins are correlated with fasting blood glucose (FBG) and the prevalence of T2D in Chinese populations. In this study, we examined the association of serum ApoA1, ApoB, and the ratio of ApoB/ApoA1 (ApoB/A1 ratio) with T2D and FBG level, and compared apolipoprotein indicators in predicting T2D in Chinese adults.

**Methods:**

A total of 1027 subjects were enrolled in this cross-sectional study. The association of ApoA1, ApoB, and ApoB/A1 ratio with T2D prevalence was determined using logistic regression models. Multivariate-analysis of covariance (ANCOVA) was performed for comparisons of the mean difference in FBG level.

**Results:**

We found that ApoB and ApoB/A1 ratio were positively associated with T2D prevalence and FBG, while inverse association was noted between ApoA1 and T2D prevalence as well as FBG. Stratified analyses for sex, age, body mass index (BMI), smoking, and alcohol consumption showed no significant difference for the association of ApoA1, ApoB, and ApoB/A1 ratio with the prevalence of T2D among subgroups (all *p*-interactions> 0.05). Nonetheless, ApoA1 poorly performed in predicting T2D as it provided an AUC value of 0.310 that was significantly lower than those observed for ApoB (AUC value: 0.631) and ApoB/A1 ratio (AUC value: 0.685). Finally, path analyses indicated that the association between ApoB and T2D was mediated by BMI.

**Conclusions:**

This study reveals the association of serum ApoA1, ApoB, and ApoB/A1 ratio with T2D and FBG in Chinese adults, suggesting that ApoB and ApoB/A1 ratio may be early indicators for predicting T2D. Prospective investigation in large cohort is needed.

**Supplementary Information:**

The online version contains supplementary material available at 10.1186/s12902-021-00726-5.

## Background

Type 2 diabetes (T2D) is a major cause of death with an estimated 5 million death worldwide in 2019 [1]. The prevalence of T2D increased 16-fold over the past 3 decades and reached 10.9% in China [1, 2]. Previous study indicated that lipoproteins exerted key effects on the pathogenesis of diabetes [3]. Therefore, the exploration of lipoprotein markers levels for T2D intervention is of high priority.

Traditional lipid concentrations are known to predict T2D, such as total cholesterol (TC), triglycerides (TG), high-density lipoprotein cholesterol (HDL-C) and low-density lipoprotein (LDL) particles [4]. There is growing interest in which lipid markers best assess diabetes risk [5–7]. Circulating apolipoproteins, which relate to the total number of lipoprotein particles, are superior to standard lipoprotein cholesterol measures for evaluating T2D risk [8, 9]. Apolipoproteins exert important roles in the metabolism of lipoprotein and blood glucose [10, 11]. Apolipoprotein A1 (ApoA1) and apolipoprotein B (ApoB) are two major types of apolipoprotein that had been well investigated. ApoA1 is the main lipoprotein associated with high-density lipoprotein cholesterol (HDL-C) [12]. ApoB is a single molecule that is presented as in low-, intermediate-, and very low-density lipoproteins [13]. In vitro and animal studies revealed that ApoA1 and ApoB modulated fasting blood glucose (FBG) level via improving insulin level [14–16]. Several studies have assessed the association of ApoA1, ApoB, and ApoB/A1 ratio with the FBG level showing inverse or null association with ApoA1 [17–19] but positive association with ApoB and ApoB/A1 ratio [11, 20, 21]. Notably, studies observed inverse and even positive association between ApoA1 and T2D presence [22–28], and positive association between T2D presence and ApoB as well as ApoB/A1 ratio [5, 23, 26, 28–30]. These results suggested that ApoA1, ApoB, and ApoB/A1 ratio may play important roles in maintaining FBG and T2D pathological process. Previous studies showed that Chinese adults had lower levels of ApoA, ApoB, and lipoprotein compared to Caucasians [31], however, little data is available for Chinese adults [24, 28] who differ from their Western counterparts in levels of lipoprotein [31]. Moreover, the different prevalence of obesity between Chinese and Western populations may have impact on the apolipoprotein-T2D association because body fat can affect the circulating glycometabolism [32, 33]. The apolipoprotein-T2D/FBG association in Chinese population remains unclear.

Obesity is an important risk factor for the pathogenesis of T2D [34]. Accumulating evidence support a significant association between circulating apolipoprotein level and obesity [35]. No study, however, investigated whether or not the association between apolipoproteins and T2D is mediated by obesity.

In this cross-sectional study, we investigated the association of ApoA1, ApoB, and ApoB/A1 ratio with T2D and FBG level, and analyzed the mediating effects of body mass index (BMI) for these associations, thus to find potential indicators for predicting T2D in Chinese population.

## Materials and methods

### Study subjects

A total of 1027 subjects including 892 non-diabetic participants and 135 T2D patients in Nantong, China, during a period from November 2018 to December 2019 were enrolled in this cross-sectional study. All subjects were > 18 years old with 590 men and 437 women. This study was approved by the Ethics Committee of Nantong Maternal and Child Healthcare Hospital (Y2018020). Written informed consent was obtained from all participants.

### Questionnaire interview and laboratory assays

Structured questionnaire [36, 37] was conducted to collect detailed sociodemographic information including sex, age, marriage, alcohol consumption (current drinker or non-drinker), smoking status (current smoker or non-smoker), exercise (light, moderate, strenuous), hypoglycemic medication and lipid-lowering drugs use by face-to-face interviews. The participants’ body height and weight were measured, and BMI was calculated. T2D was identified by FBG level (≥ 7.0 mmol/L), self-report, and relevant treatments. Blood samples were obtained from the participants after a 10-h fast. Serum TC and TG were measured using the Denka Seiken enzymatic colorimetric test (Tokyo, Japan). HDL-C and LDL-C were measured using commercial reagents (Beckman Coulter Inc., Brea, CA). The coefficients of variation (CVs) for TC, TG, HDL-C and LDL-C were 2.1, 1.5, 3.7 and 4.1%, respectively. FBG was measured using a commercial kit (Roche Diagnostics GmbH, China). The CV of FBG was 2.7%.

Serum ApoA1 and ApoB concentrations were determined according to standardized operation. Briefly, 3 μL serum sample were mixed with 240 μL reagent composed of phosphate buffer (0.05 mol/L, pH = 7.0) and polyethylene glycol (30 g/L). The mixture was vortexed for 2 min and then incubated at 37 °C for 5 min. The lyophilized products containing ApoA1 or ApoB were used as calibrator. The polyethylene glycol-enhanced immunology turbidimetric assay was conducted to detect the concentrations of ApoA1 and ApoB in a 7600–010 automatic analyzer (Hitachi, Japan). The intra-assay CVs of ApoA1 and ApoB were 3.2 and 4.9%, respectively. The inter-assay CVs of ApoA1 and ApoB were 4.7 and 5.1%, respectively. ApoB/A1 ratio was calculated.

### Statistical analyses

The baseline characteristics of the participants were described by means with standard deviations (SD) for the continuous variables and frequencies (percentages) for the categorical variables stratified by T2D status (Yes/No). Student’s t-test or the Mann–Whitney U test was used to compare the continuous variables and Chi-square test for the comparisons of the categorical variables. The subjects were categorized into quartiles according to ApoA1, ApoB, and ApoB/A1 ratio, respectively. Logistic regression model was used to estimate the association of ApoA1, ApoB, and ApoB/A1 ratio with the prevalence of T2D. Odds ratios (ORs) and 95% confidence intervals (CIs) of the T2D for the 2nd – 4th quartiles of apolipoprotein concentrations were computed, with the lowest quartile defined as the reference. Multivariate-analyses of covariance (ANCOVAs) were used to compare the mean differences of FBG level and test trends in quartiles of apolipoproteins. The Bonferroni test was performed for pair-wise comparisons among quartiles. In Model 1, age was adjusted. In Model 2, sex, age, marriage, alcohol consumption, smoking status, exercise, and hypoglycemic drug use were adjusted. In Model 3, lipid-lowering drugs use was further adjusted. Stratified analyses were performed by sex (Men vs. Women), age (≤ 60 vs. > 60 years), BMI (≤ 24 vs. > 24 kg/m^2^), smoking (Yes vs. No) and drinking (Yes vs. No). Multiplicative interactions were assessed through likelihood ratio test. The receiver-operating characteristic (ROC) curves were drawn and values of area under curve (AUC) were estimated. Path analyses were conducted to assess the mediating effects of BMI on apolipoproteins-T2D association [38]. Two parts of path analyses were performed: one related to the association of apolipoproteins with BMI, the other related to the association of BMI with T2D. Standardized regression coefficients were obtained to evaluate correlation in each path. Two-tailed *p* < 0.05 was considered statistically significant. The analyses were performed using SPSS Statistics 21.0 (IBM SPSS Statistics, Inc., Armonk, NY, USA). Path analyses were conducted using SPSS AMOS21.0 (IBM Corporation, Armonk, NY, USA).

## Results

### Characteristics of the study subjects

As shown in Table 1, the mean age of the T2D subjects and non-T2D participants was 63.6 and 59.8 years, respectively. Participants with T2D exhibited significantly higher BMI (24.6 kg/m^2^ vs. 23.9 kg/m^2^), FBG level (8.95 mmol/L vs. 5.36 mmol/L), TC (5.79 mmol/L vs. 5.58 mmol/L), TG (1.92 mmol/L vs. 1.58 mmol/L), ApoB (1.29 g/L vs. 1.14 g/L), and ApoB/A1 ratio (1.88 vs. 1.08) and lower HDL-C (1.24 mmol/L vs. 1.34 mmol/L) and ApoA1 (0.85 g/L vs. 1.21 g/L) compared to non-diabetic participants (all *p* < 0.05).

**Table 1:** Characteristics of the study participants

Variables	T2D (*n* = 135)	Non-T2D (*n* = 892)	*p*
Age, years	63.6 ± 13.5	59.8 ± 10.5	**< 0.001**
Female, n (%)	63 (41.93)	374 (46.67)	0.299
BMI, kg/m^2^	24.6 ± 3.3	23.9 ± 5.5	**0.047**
Marriage, n (%)			0.584
Married	123 (91.1)	818 (91.7)	
Unmarried	1 (0.7)	15 (1.7)	
Divorce/Widowed	11(8.2)	59 (6.6)	
Education level, n (%)			**0.033**
Junior high school	100 (74.1)	605 (67.8)	
High school	26 (19.3)	153 (17.2)	
College degree or above	9 (6.6)	134 (15.0)	
Smoker, n (%)	25 (18.5)	189 (21.2)	0.856
Alcohol drinker, n (%)	57 (42.2)	384 (43.0)	0.477
Hypoglycemic drugs use, n (%)	43 (31.9)	0 (0.0)	**< 0.001**
Lipid-lowering drugs use, n (%)	12 (8.1)	48 (5.4)	0.105
Exercise, n (%)			0.455
Sitting	58 (43.0)	343 (38.5)	
Light	57 (42.2)	366 (41.0)	
Moderate	15 (11.1)	141 (15.8)	
Strenuous	5 (3.7)	42 (4.7)	
FBG, mmol/L	8.95 ± 1.65	5.36 ± 0.66	**< 0.001**
TC, mmol/L	5.79 ± 1.09	5.58 ± 1.16	**0.043**
TG, mmol/L	1.92 ± 1.10	1.58 ± 1.06	**< 0.001**
HDL-C, mmol/L	1.24 ± 0.31	1.34 ± 0.34	**0.002**
LDL-C, mmol/L	3.58 ± 0.89	3.54 ± 0.96	0.620
Apolipoproteins, g/L			
ApoA1	0.85 ± 0.58	1.21 ± 0.40	**< 0.001**
ApoB	1.29 ± 0.34	1.14 ± 0.33	**< 0.001**
ApoB/A1 ratio	1.88 ± 1.30	1.08 ± 0.64	**< 0.001**

*BMI* body mass index, *FBG* fasting blood glucose, *Apo* apolipoprotein, *TC* total cholesterol, *TG* triglyceride, *HDL-C* high-density lipoprotein cholesterol, *LDL-C* low-density lipoprotein cholesterol, *T2D* type 2 diabetes

### Association of ApoA1, ApoB, and ApoB/A1 ratio with T2D prevalence

We initially analyzed whether or not serum ApoA1, ApoB, and ApoB/A1 ratio were associated with T2D prevalence and found a trend that serum ApoA1 was inversely associated with T2D prevalence. In contrast, ApoB and ApoB/A1 ratio were positively associated with T2D prevalence in three models (Table 2). In Model 1 with adjustment for age, the ORs (95% CI) in the fourth quartile were 0.29 (0.18, 0.46) for inverse association of serum ApoA1, 3.09 (1.71, 5.55) for positive association of ApoB, and 5.12 (2.91, 9.02) for positive association of ApoB/A1 ratio compared with the first quartile, respectively. In Model 2 with adjustment for other potential covariates, inverse associations for ApoA1 were found with ORs (95% CI) of 0.15 (0.07, 0.33) in the second quartile, and of 0.37 (0.20, 0.68) in the third quartile compared with the first quartile, respectively. In contrast, positive associations for ApoB and ApoB/A1 ratio were found with ORs (95% CI) of 3.52 (1.67, 7.41) and 2.53 (1.35, 4.73) in the fourth quartile compared to first quartile, respectively (*p*-trend: < 0.001–0.023). After further adjusting for lipid-lowering drugs use in Model 3, the prevalence of T2D in the second and third quartiles of ApoA1 decreased 85% (OR = 0.15, 95% CI: 0.07, 0.34) and 63% (OR = 0.37, 95% CI: 0.20, 0.68) compared to the first quartile, respectively. The multivariable-adjusted ORs (95% CI) of the T2D presence for the fourth quartile were 3.38 (1.60, 7.14) for ApoB and 2.39 (1.27, 4.52) for ApoB/A1 ratio compared to first quartile, respectively (*p*-trend: < 0.001–0.036). In stratified analyses by sex, age, BMI, smoking, and drinking, the association of ApoA1, ApoB, and ApoB/A1 ratio with T2D prevalence was not significantly different among subgroups (all *p*-interactions > 0.05, Table 3 and Supplementary Table 1).

**Table 2:** The associations of ApoA1, ApoB levels and ApoB/A1 ratio with T2D prevalence

Variables	Quartiles by Apolipoproteins				*p*
	Q1	Q2	Q3	Q4	
ApoA1, g/L					
Mean (SD)	0.59 (0.20)	0.99 (0.10)	1.34 (0.10)	1.73 (0.16)	
Case/N	76/255	10/255	20/257	29/258	
OR (95% CI)					
Model 1	1	0.09 (0.05, 0.18)^**^	0.20 (0.12, 0.35)^**^	0.29 (0.18, 0.46)^**^	**< 0.001**
Model 2	1	0.15 (0.07, 0.33)^**^	0.37 (0.20, 0.68)^**^	0.60 (0.35, 1.02)	0.232
Model 3	1	0.15 (0.07, 0.34)^**^	0.37 (0.20, 0.68)^**^	0.60 (0.35, 1.03)	0.232
ApoB, g/L					
Mean (SD)	0.77 (0.14)	1.03 (0.06)	1.23 (0.06)	1.60 (0.25)	
Case/N	17/262	25/252	46/260	47/253	
OR (95% CI)					
Model 1	1	1.53 (0.80, 2.91)	2.91 (1.62, 5.24)^**^	3.09 (1.71, 5.55)^**^	**< 0.001**
Model 2	1	1.68 (0.75, 3.79)	3.59 (1.72, 7.49)^**^	3.52 (1.67, 7.41)^**^	**< 0.001**
Model 3	1	1.65 (0.73, 3.73)	3.49 (1.67, 7.31)^**^	3.38 (1.60, 7.14)^**^	**< 0.001**
ApoB/A1 ratio					
Mean (SD)	0.58 (0.08)	0.79 (0.07)	1.10 (0.13)	2.26 (0.95)	
Case/N	17/256	31/257	16/258	71/256	
OR (95% CI)					
Model 1	1	1.82 (0.98, 3.38)	0.84 (0.41, 1.71)	5.12 (2.91, 9.02)^**^	**< 0.001**
Model 2	1	1.56 (0.82, 2.97)	0.75 (0.36, 1.58)	2.53 (1.35, 4.73)^**^	**0.023**
Model 3	1	1.50 (0.78, 2.87)	0.72 (0.34, 1.52)	2.39 (1.27, 4.52)^*^	**0.036**

Model 1 was adjusted for age. Model 2 was adjusted for sex, age, BMI, education, marriage, exercise, cigarette smoking, alcohol consumption and hypoglycemic drugs use. Model 3 further adjusted for lipid-lowering drugs use

*Apo* apolipoprotein, *SD* standard deviation, *OR* odds ratio, *95%CI* 95% confidence interval, *Q* quartile, *T2D* type 2 diabetes

^*^p < 0.05 compared with Q1

^**^p < 0.001 compared with Q1

**Table 3:** The associations of ApoA1, ApoB levels and ApoB/A1 ratio with T2D prevalence by sex (OR, 95% CI)

Variables	Quartiles by Apolipoproteins				*p*-interaction
	Q1	Q2	Q3	Q4	
ApoA1					0.136
Men	1	0.32 (0.12, 0.86)^*^	0.74 (0.33, 1.65)	0.98 (0.45, 2.11)	
Women	1	0.05 (0.01, 0.22)^**^	0.14 (0.05, 0.40)^**^	0.38 (0.17, 0.86)^*^	
ApoB					0.727
Men	1	1.24 (0.44, 3.51)	2.44 (0.97, 6.16)	2.59 (1.02, 6.58)^*^	
Women	1	2.74 (0.69, 10.82)	5.56 (1.53, 20.25)^*^	4.46 (1.19, 16.70)^*^	
ApoB/A1 ratio					0.792
Men	1	1.42 (0.59, 3.44)	0.92 (0.36, 2.38)	2.09 (0.87, 5.01)	
Women	1	1.18 (0.44, 3.19)	0.32 (0.09, 1.17)	2.53 (0.95, 6.74)	

Age, BMI, education, marriage, exercise, cigarette smoking, alcohol consumption, hypoglycemic drugs use, and lipid-lowering drugs use were adjusted

*Apo* apolipoprotein, *OR* odds ratio, *95%CI* 95% confidence interval, *Q* quartile, *T2D* type 2 diabetes

^*^p < 0.05 compared with Q1

^**^p < 0.001 compared with Q1

### Association of lipid parameters with T2D prevalence

Among lipid parameters, HDL-C was inversely associated with T2D prevalence, but positive associations of TG and LDL-C with T2D prevalence were detected across the three models. Null association was found between TC and T2D prevalence in multivariate analysis (Supplementary Table 2).

### Association of ApoA1, ApoB, and ApoB/A1 ratio with FBG level

In three Models, serum ApoA1 exhibited an inverse association with FBG, while ApoB and ApoB/A1 ratio showed positive association with FBG (Table 4). With adjustment for age in Model 1, lower FBG level was associated with higher ApoA1 concentration. In contrast, increased FBG level was detected in subjects with higher ApoB level and ApoB/A1 ratio. With adjustment for the other potential covariates in Model 2, intermediate ApoA1 concentration was negatively associated with FBG level (Q2 vs. Q1 = 5.574 mmol/L vs. 6.067 mmol/L; Q3 vs. Q2 = 5.881 mmol/L vs. 5.574 mmol/L). In contrast, ApoB and ApoB/A1 ratio were positively associated with FBG level (Q4 vs. Q1 = 5.910 mmol/L vs. 5.728 mmol/L for ApoB; Q3 vs. Q2 = 5.679 mmol/L vs. 5.972 mmol/L for ApoB/A1 ratio). After further adjusting for lipid-lowering drugs use in Model 3, higher ApoA1 concentration were associated with significantly lower FBG level (Q2 vs. Q1 = 5.575 mmol/L vs. 6.067 mmol/L). In contrast, positive association between ApoB and ApoB/A1 ratio and FBG level were shown. The corresponding FBG values were 5.908 mmol/L (vs. 5.730 mmol/L) for ApoB in the fourth quartile compared with the first quartile, and 5.678 mmol/L (vs. 5.971 mmol/L) for ApoB/A1 ratio in the third quartile compared with the second quartile.

**Table 4:** Mean FBG level according to quartiles of ApoA1, ApoB levels and ApoB/A1 ratio

Variables	Quartiles by Apolipoproteins				*p*-Diff	*p*-Trend
	Q1	Q2	Q3	Q4		
ApoA1						
n	255	257	257	258		
FBG, mmol/L						
Model 1	6.891 ± 0.113	5.316 ± 0.113^**^	5.636 ± 0.113^**^	5.802 ± 0.113^**#^	**< 0.001**	**< 0.001**
Model 2	6.067 ± 0.078	5.574 ± 0.074^**^	**5.881 ± 0.074**^**#**^	**6.117 ± 0.074**^**##**^	**< 0.001**	0.184
Model 3	6.067 ± 0.078	5.575 ± 0.074^**^	**5.880 ± 0.075**^**#**^	**6.117 ± 0.074**^**##**^	**< 0.001**	0.184
ApoB						
n	262	252	260	253		
FBG, mmol/L						
Model 1	5.637 ± 0.117	5.842 ± 0.119	6.136 ± 0.117^*^	6.026 ± 0.119	**0.017**	**0.006**
Model 2	5.728 ± 0.074	5.914 ± 0.075	6.087 ± 0.074^*^	5.910 ± 0.075	**0.009**	**0.034**
Model 3	5.730 ± 0.075	5.914 ± 0.075	6.087 ± 0.074^*^	5.908 ± 0.076	**0.010**	**0.038**
ApoB/A1 ratio						
n	256	257	258	256		
FBG, mmol/L						
Model 1	5.622 ± 0.115	5.787 ± 0.114	5.419 ± 0.114	6.814 ± 0.114^**#¶^	**< 0.001**	**< 0.001**
Model 2	5.906 ± 0.076	5.972 ± 0.074	**5.679 ± 0.075**^**#**^	**6.083 ± 0.077**^**¶**^	**0.002**	0.499
Model 3	5.910 ± 0.077	5.971 ± 0.074	**5.678 ± 0.075**^**#**^	**6.080 ± 0.078**^**¶**^	**0.002**	0.533

Model 1 was adjusted for age. Model 2 was adjusted for sex, age, BMI, education, marriage, exercise, cigarette smoking, alcohol consumption and hypoglycemic drugs use. Model 3 further adjusted for lipid-lowering drugs use

*Apo* apolipoprotein, *FBG* fasting blood glucose, *Q* quartile. *p*-Diff: Multiple comparison among quartiles

^*^p < 0.05 compared with Q1

^**^p < 0.001 compared with Q1

^#^*p* < 0.05 compared with Q2

^##^*p* < 0.001compared with Q2

^¶^*p* < 0.05 compared with Q3

### Performance of apolipoprotein indicators

AUC was used to evaluate the performance of ApoA1, ApoB, and ApoB/A1 ratio for predicting T2D (All *p* < 0.001). As shown in Table 5, ApoA1 poorly performed at predicting T2D with an AUC value of 0.310 that was significantly smaller than AUCs observed from ApoB (AUC value: 0.631) and ApoB/A1 ratio (AUC value: 0.685). The corresponding ROC curves were shown in Supplementary Fig. 1, 2 and 3.

**Table 5:** The ROC curves parameters for ApoA1, ApoB levels and ApoB/A1 ratio in predicting T2D

Variables	AUC	95% CI	Sensitivity (%)	Specificity (%)	*p* value
ApoA1	0.310	0.249, 0.371	0.156	0.846	**< 0.001**
ApoB	0.631	0.584, 0.679	0.622	0.611	**< 0.001**
ApoB/A1 ratio	0.685	0.632, 0.739	0.489	0.861	**< 0.001**

*Apo* apolipoprotein, *AUC* area under curve, *ROC* receivers operator characteristic, *T2D* type 2 diabetes, *95%CI* 95% confidence interval

### Path analyses

**Fig. 1:**
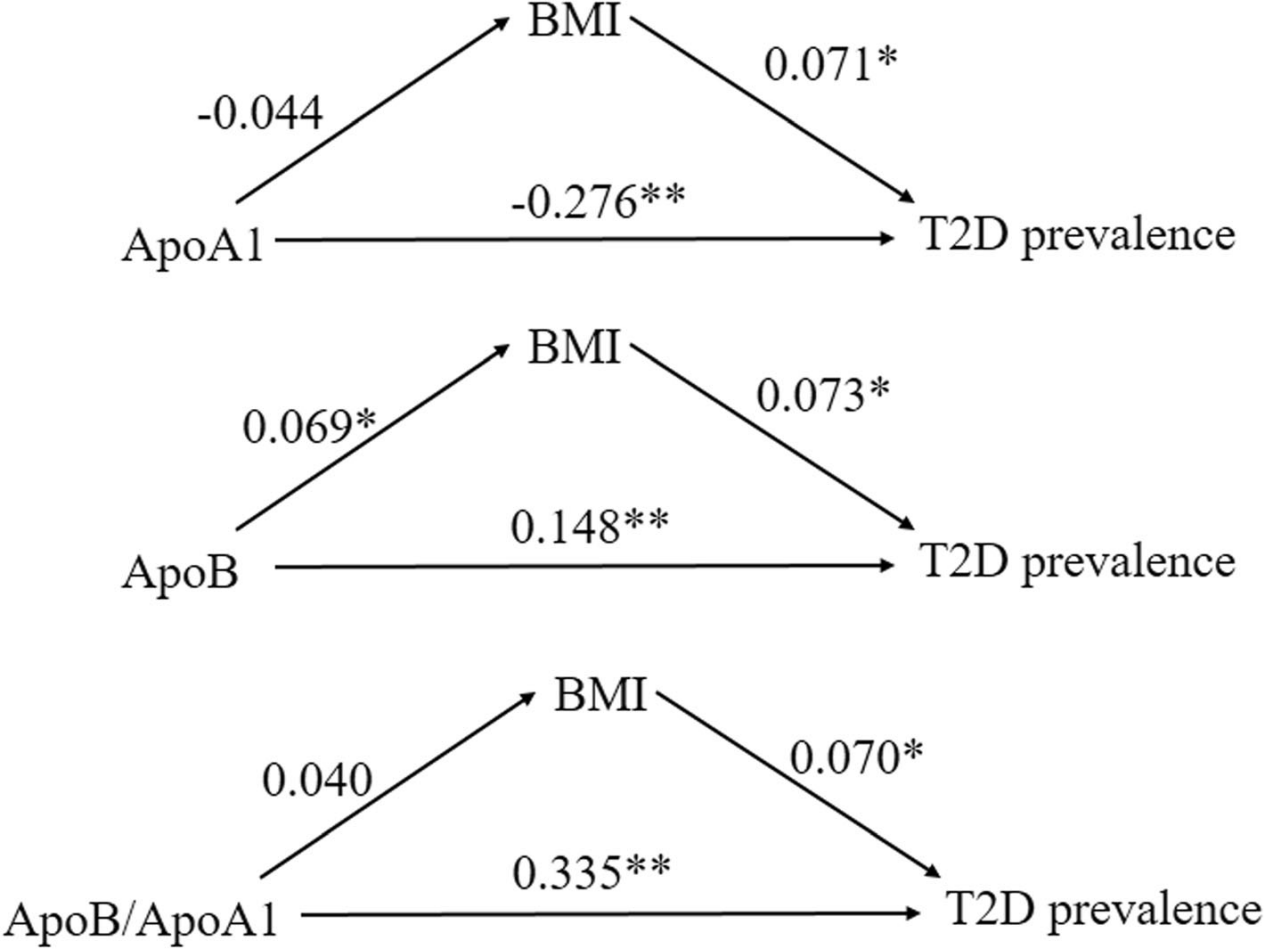
Path analyses of associations of ApoA1, ApoB levels, ApoB/A1 ratio, and mediators (BMI) with the prevalence of T2D in Chinese adults, respectively. Apo, apolipoprotein; BMI, body mass index; T2D, Type 2 diabetes. * *p* < 0.05; ** *p* < 0.001

Path analyses indicated that ApoB-T2D prevalence association was mediated by BMI (Fig. 1). BMI was positively correlated to increased serum ApoB level (r = 0.069, *p* < 0.05) and T2D prevalence (r = 0.073, *p* < 0.05). Serum ApoA1 and ApoB/A1 ratio had no direct effect on BMI (all *p* > 0.05). No mediating effect of BMI on the association of ApoA1-T2D and ApoB/A1–T2D was found.

## Discussion

In this community-based cross-sectional study, we estimated the association of serum levels of ApoA1 and ApoB as well as the ratio of ApoB/A1 with T2D prevalence and FBG level. We found that serum ApoA1 was favorably associated with a decreased prevalence of T2D, whereas ApoB and ApoB/A1 ratio were positively associated with T2D prevalence. Consistent with these findings, we observed an inverse association of serum ApoA1 with FBG, and positive association of ApoB and ApoB/A1 ratio with FBG. Moreover, ApoB and ApoB/A1 ratio performed better than ApoA1 in predicting T2D prevalence in a Chinese population.

ApoA1, a major protein component of HDL with anti-inflammatory and immunomodulatory effect [12, 39], is found in significant amounts on chylomicron remnants [40]. The protective effect of HDL is due to lipoprotein(a) particles, especially ApoA1 [41]. Our results demonstrated that both ApoA1 and HDL-C were inversely associated with the T2D prevalence and an intermediate ApoA1 concentration showed a lower T2D prevalence compared with HDL-C. Similarly, ApoA1 has been observed to be equivalent or better than HDL-C as a risk marker for cardiovascular or atherosclerosis events [42, 43]. Previous studies reported that ApoA1 had cardio- and bone mass-protective roles [39, 44]. Although ApoA1 can modulate FBG, the association of ApoA1 with T2D prevalence and FBG level in Chinese populations is ill-characterized. Based on data from 1514 males participated in the ATTICA 2001–2012 surveys, the 10-year T2D risk decreased 1.02% with every 1 mg/dL increase of apoA1 [22]. FINRISK97 cohort consisted of 7827 middle aged persons found that ApoA were significantly associated with incident diabetes in Finland [45]. Wu et al. observed that low level of ApoA1 was independently associated with T2D (OR: 0.51, 95% CI: 0.33, 0.76) [24]. TwSHHH indicated the inverse association between ApoA1 and incident T2D among 4223 adults in Taiwan, Chinese (hazard ratio [HR]: 0.59, 95% CI: 0.38, 0.92) [28]. But fasting plasma glucose level was adjusted in multivariate analysis of this prospective study, which might be more likely to obtain inverse associations. Similar results were also reported by other groups [23, 25, 26]. Consistent with these findings, we observed a beneficial effect of ApoA1 on T2D presence. In contrast, null and even positive association was observed in western population [22, 27, 46]. Mellor et al. reported that serum ApoA1 was not associated with the risk of diabetes in a prospective cohort composed of 759 females in Greek population [22]. In a 13.5 years prospective study with 110 incident T2D and 741 non-T2D subjects within the Rotterdam Study, Brahimaj et al. [46] found null association between serum ApoA1 and T2D risk in the Dutch population. Onat et al. even found the positive association of serum ApoA1 with diabetes in Turkish population (OR: 1.85, 95% CI: 1.03, 3.32) [27]. In addition, several studies assessed the association of ApoA1 with FBG level showing inconsistent results (inverse or null association) [18, 19, 47]. Similar to our results, previous studies found plasma glucose levels were negatively correlated to ApoA1 in Chinese and Dutch population (r: − 0.1182 and − 0.013) [18, 46]. The heterogeneity among different studies can be caused by different mechanisms. Significant results were more likely to be obtained in cross-sectional study (vs. prospective study [22]). The lower ApoA1 concentration that was found in The Rotterdam Study (7.4 μmol/L) [46] than in the present study (39.0 μmol/L) might provide a potential explanation for the null findings of the prior one study. ApoA1 might turn to enhance risk of diabetes due to dysfunctional adiponect in prevailing among Turks (vs. this study) [27]. Moreover, favorable association is readily to be found in large sample size, non-obesity population, and Asian population as observed in the present study and previously reported. Although we were unable to characterize the shape of the exposure–outcome relation due to the limited number of samples, our results implied that an intermediate ApoA1 level was important in FBG regulation. Large prospective study is needed to confirm this speculation.

A series of studies have shown the association of serum ApoB and ApoB/A1 ratio with T2D prevalence and FBG level [5, 7, 20–23, 27, 28, 30, 48]. A large multiethnic cohort study of kidney transplant recipients found that serum ApoB and ApoB/A1 ratio were positively associated with post-transplantation diabetes mellitus with an OR of 12.2 (95%CI: 3.13, 47.5) for ApoB and OR of 8.25 (95%CI: 1.72, 39.5) for ApoB/A1 ratio, respectively [23]. A retrospective longitudinal study in Korea also observed that ApoB level and ApoB/A1 ratio were positively associated with T2D, the OR values were 1.262 (95%CI: 1.107, 1.438) for ApoB and 1.292 (95%CI: 1.137, 1.468) for ApoB/A1 ratio, respectively [5]. TwSHHH study also pointed out the risk of T2D associated with serum levels of ApoB and ApoB/A1 ratio among adults in Taiwan, China (OR _ApoB_: 2.21, 95% CI _ApoB_: 1.12, 4.16; OR _ApoB/A1_: 2.46, 95% CI _ApoB/A1_: 1.32, 4.58) [28]. But fasting plasma glucose level was adjusted in this prospective study. In addition, a cross-sectional study with 70,063 subjects found a positive association of ApoB/A1 ratio with T2D (OR: 1.31, 95%CI: 1.17, 1.46) [30]. Similar to these findings, the present study found positive association of ApoB level and ApoB/A1 ratio with T2D prevalence. Our study also confirmed the positive association of ApoB and ApoB/A1 ratio with FBG level as previously reported [20, 21]. However, the Atherosclerosis Risk in Communities (ARIC) study of 9026 American populations reported the null effects of ApoB and ApoB/A1 ratio on diabetes [7]. Analogous findings were seen in Turkish population [27], Australian women with previous gestational diabetes mellitus [48] and Greek population [22]. The inconsistent results of the present and previous studies might be attributable to some factors. Interestingly, the positive association was more likely to be observed in persons with higher ApoB concentration ([116 mg/dL] for this study) than lower concentration (99.58 mg/dL for American [7] and 106.36 mg/dL for Greece [22]), in studies with relative large sample size ([5, 23, 30] & this study vs. small sample size [48]). Additionally, other reasons such as the differences in design of study (cross-sectional vs. prospective), different obesity (Chinese vs. Western population), and covariates that were adjusted might also provide an explanation for the inconsistent findings among these studies.

It is worth noting that ApoB and ApoB/A1 ratio showed higher OR for T2D, compared with the TC, TG and LDL-C. These findings were supported by previous study results. An aboriginal Canadian study indicated that plasma ApoB concentration was positively associated with T2D risk and was superior to LDL-C in predicting the disease in aboriginal Canadian population [49]. Additionally, ApoB showed a stronger association with the incident T2D compared with conventional lipid measurements in Korean populations [50]. Previous study revealed that ApoB/A1 ratio added significant information for predicting insulin resistance [51]. Indeed, ApoB was contained in each of very low LDL, intermediate-density lipoprotein, LDL-C, and lipoprotein(a) particles [8]. Taken together, these finds suggested that Apo levels either separately for ApoB or together as calculated ApoB/A1 ratio might provide additional information for predicting the incident T2D.

### Mechanisms

The association of serum ApoA1, ApoB, and ApoB/A1 ratio with T2D may be through various mechanisms. First, ApoA1 can reduce lipid binding capability, alter protein structure, and attenuate ability of catalyzing cholesterol efflux from macrophages [17]. In addition, ApoA1 is able to improve glucose tolerance via increasing glucose uptake into skeletal muscle and heart by adenosine monophosphate-activated protein kinasecomplex [52]. Second, ApoB inhibits lipolysis in adipocytes by acting as a metabolic pathway from liver to peripheral fat [53]. Dysregulation of ApoB metabolism can consequently cause insulin resistance [54]. Furthermore, path analyses in the present study confirmed an impact of ApoB on BMI. Third, oxidative stress and inflammation exert important roles in T2D pathophysiology. Several studies have demonstrated that ApoA1 protects mice against inflammation and oxidative stress-induced damage via clearing pro-inflammatory lipids and decreasing plasma malondialdehyde levels and intestinal inflammation in a Cyclooxygenase 2 total knockout and myeloid knockout/ cholate-containing high fat diet model [55, 56]. Finally, ApoB can aggravate inflammation by binding to enolase-1 and releasing more inflammatory cytokines (e.g., tumor necrosis factor-α, IL-1β, and IL-6) [57].

### Strengths and limitations

This study comprehensively assessed the association of ApoA1, ApoB, and ApoB/A1 ratio with T2D prevalence and FBG level. Importantly, to the best of our knowledge, this is the first report that analyzes the mediating effects of BMI on the association of ApoA1, ApoB, and ApoB/A1 ratio with T2D prevalence in Chinese adults. Next, the relatively large sample size allows us to be able to evaluate potential association among variables. Third, the availability of individual information (e.g., medication records and lifestyles) allows us to adjust for more potential confounders. Fourth, we further evaluated the effect of the interactions between ApoA1, ApoB, ApoB/A1 ratio and individual information (e.g., sex, age, BMI, smoking, and drinking status) on T2D prevalence. Finally, by comparing apolipoprotein indicators, we found that ApoB and ApoB/A1 ratio performed better in predicting T2D, which may provide specific guidance for large epidemiological surveys for T2D. Notwithstanding, the present study also has a few limitations. First, the causality was not determined due to the cross-sectional study design. However, apolipoprotein levels reached a steady-state in adults, and this state was mainly influenced by diets rather than glucose metabolism indices. Therefore, cause-and-effect association is less likely to be inverted. Second, although all subjects were recruited from urban communities in China, it had little influence on the association of ApoA1, ApoB and ApoB/A1 ratio with T2D prevalence or FBG level. Third, previous study reported dietary nutrients were related to Apo concentrations [58], however, we did not rule out the effects of dietary nutrients, which might attenuate the underlying associations. Fourth, although daily physical activity was assessed by a physical activity questionnaire, subjective recall bias and day-to-day fluctuations from daily physical activity in our study were possible, but which might have minimal impact on the present results due to daily physical activity was relative stable in participants with an average age of 60.3 years. Finally, although attempts to control for the potential confounders have been made, residual confounding may not be ruled out as reported by other observational studies.

## Conclusion

In summary, this study provides support for the modulating effect of serum ApoA1, ApoB, and ApoB/A1 ratio on T2D prevalence and FBG level. Of these, ApoB and ApoB/A1 ratio are appropriate apolipoprotein indicators for predicting T2D in Chinese adults. Replication of these findings in large prospective studies is anticipated.

## Supplementary Information

**Additional file 1: Supplementary Figure 1.** The ROC curves parameters for Apo A1 level in predicting T2D. ROC, Receivers Operator Characteristic; Apo, Apolipoprotein; T2D, Type 2 diabetes.

**Additional file 2: Supplementary Figure 2.** The ROC curves parameters for ApoB level in predicting T2D. Abbreviations were showed in Supplementary Fig. 1.

**Additional file 3: Supplementary Figure 3.** The ROC curves parameters for ApoB/A1 ratio in predicting T2D. Abbreviations were showed in Supplementary Fig. 1.

**Additional file 4: Supplementary Table 1.** The multivariable associations of ApoA1, ApoB levels and ApoB/A1 ratio with T2D prevalence by subgroups (OR, 95% CI). **Supplementary Table 2** The associations of TC, TG, HDL and LDL levels with T2D prevalence (OR, 95% CI).

## Data Availability

The datasets used and/or analyzed during the current study available from the corresponding author on reasonable request.

## Abbreviations

ANCOVAs: Analyses of covariance
ApoA1: Apolipoprotein A1
ApoB: Apolipoprotein B
AUC: Area under curve
BMI: Body mass index
CIs: Confidence intervals
CV: Coefficient of variation
FBG: Fasting blood glucose
HDL-C: High-density lipoprotein cholesterol
HR: Hazard ratio
IL: Interleukins
LDL-C: Low-density lipoprotein cholesterol
ORs: Odds ratios
ROC: Receiver-operating characteristic curves
SD: Standard deviation
T2D: Type 2 diabetes
TC: Total cholesterol
TG: Triglyceride
