# Supplementary figures and images for: Association of apolipoproteins A1 and B with type 2 diabetes and fasting blood glucose: a cross-sectional study
Source: BMC Endocr Disord. 2021 Apr 1;21:59. doi: 10.1186/s12902-021-00726-5 (PMC8017773; doi:10.1186/s12902-021-00726-5)

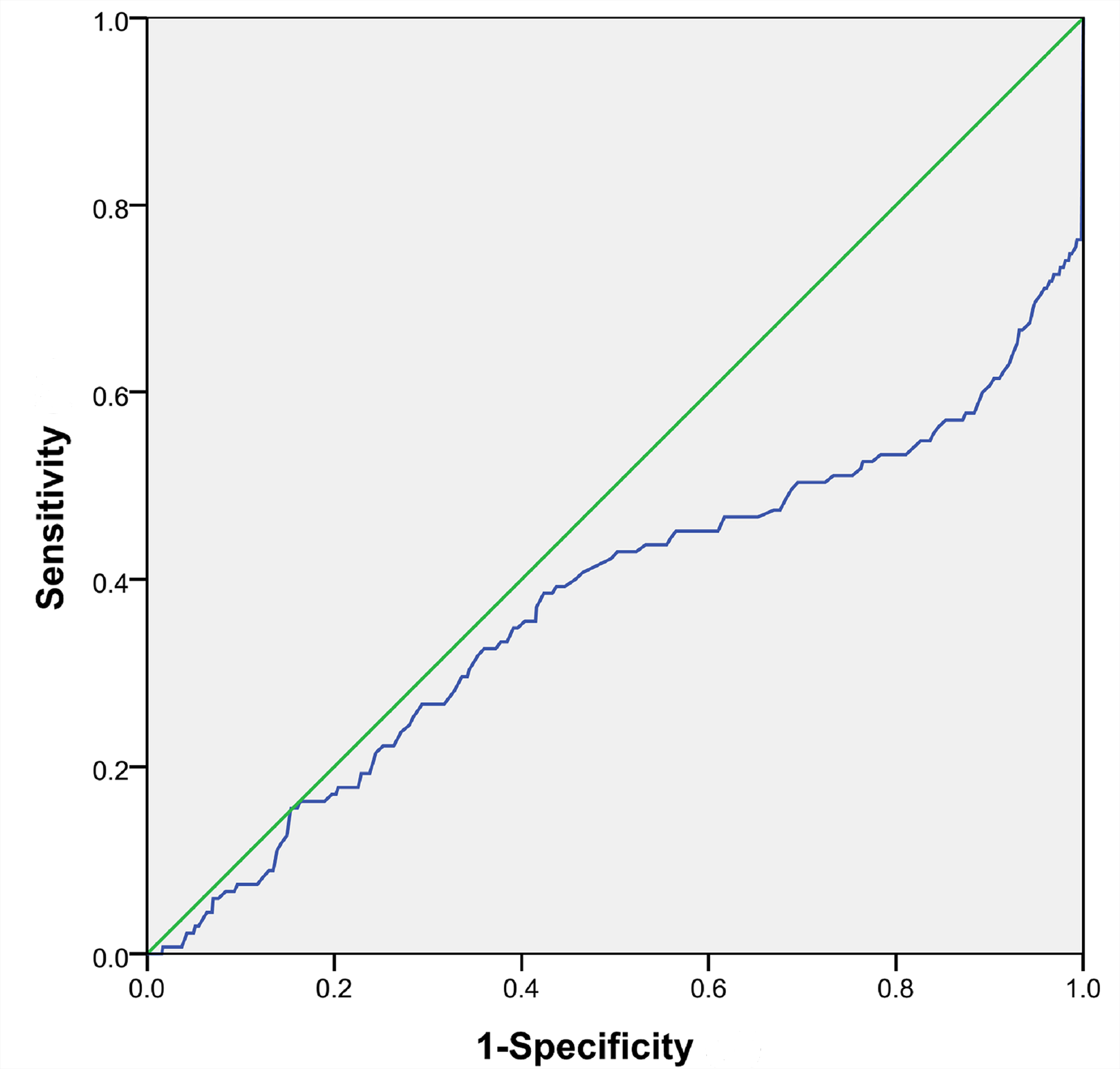

Supplement: Supplementary file 1 — Additional file 1: Supplementary Figure 1. The ROC curves parameters for Apo A1 level in predicting T2D. ROC, Receivers Operator Characteristic; Apo, Apolipoprotein; T2D, Type 2 diabetes. [file 12902_2021_726_MOESM1_ESM.tif]

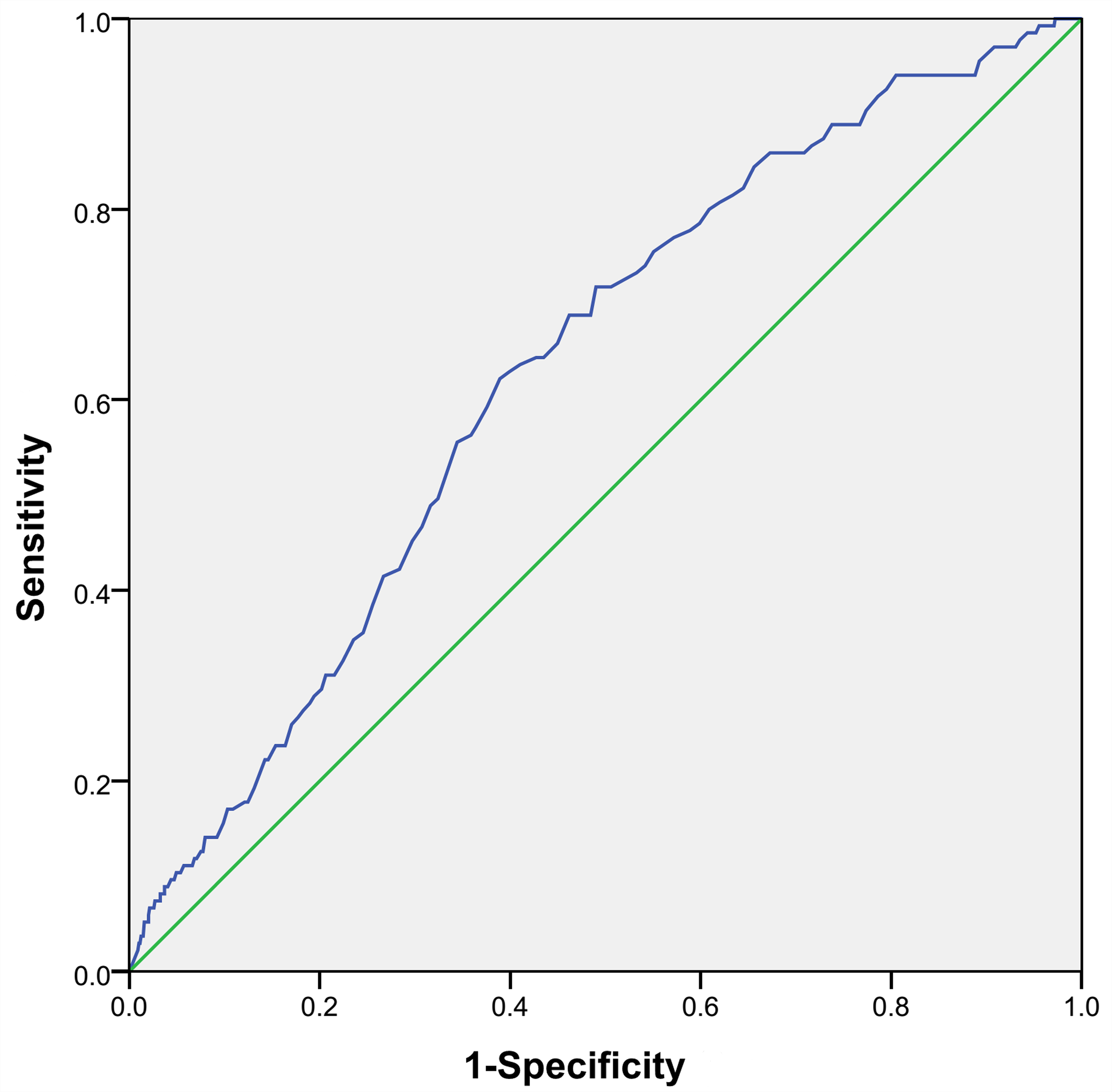

Supplement: Supplementary file 2 — Additional file 2: Supplementary Figure 2. The ROC curves parameters for ApoB level in predicting T2D. Abbreviations were showed in Supplementary Fig. 1. [file 12902_2021_726_MOESM2_ESM.tif]

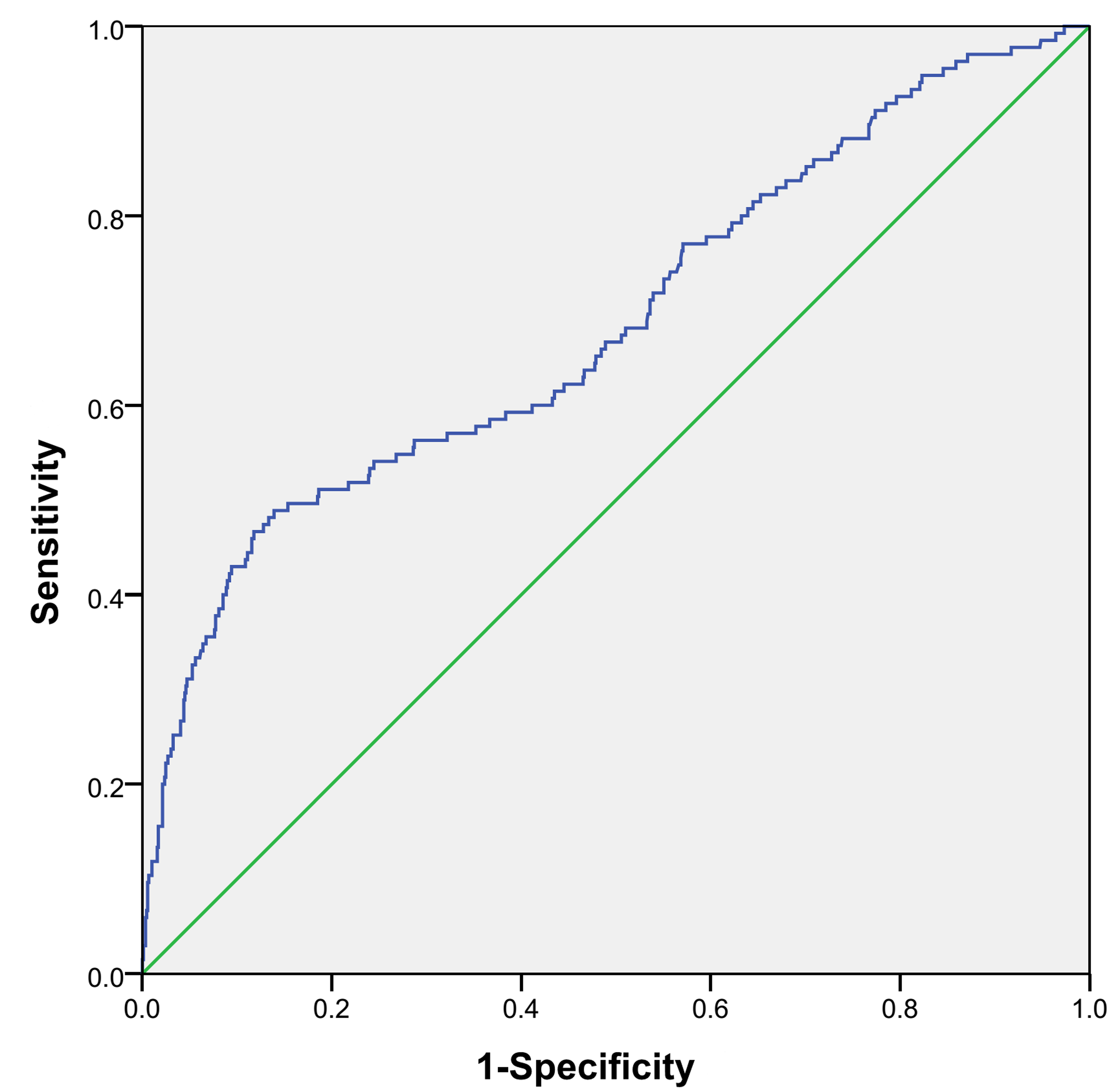

Supplement: Supplementary file 3 — Additional file 3: Supplementary Figure 3. The ROC curves parameters for ApoB/A1 ratio in predicting T2D. Abbreviations were showed in Supplementary Fig. 1. [file 12902_2021_726_MOESM3_ESM.tif]
