# Supplementary material for: Association of apolipoproteins A1 and B with type 2 diabetes and fasting blood glucose: a cross-sectional study
Source: BMC Endocr Disord. 2021 Apr 1;21:59. doi: 10.1186/s12902-021-00726-5 (PMC8017773; doi:10.1186/s12902-021-00726-5)
Supplement: Supplementary file 4 — Additional file 4: Supplementary Table 1. The multivariable associations of ApoA1, ApoB levels and ApoB/A1 ratio with T2D prevalence by subgroups (OR, 95% CI). Supplementary Table 2 The associations of TC, TG, HDL and LDL levels with T2D prevalence (OR, 95% CI). [file 12902_2021_726_MOESM4_ESM.docx]

**Supplementary Table 1** The multivariable associations of ApoA1, ApoB levels and ApoB/A1 ratio with T2D prevalence by subgroups (OR, 95% CI).

| Variables | Quartiles by Apolipoproteins | | | | *p*-interaction |
| --- | --- | --- | --- | --- | --- |
|  | Q1 | Q2 | Q3 | Q4 |  |
| ApoA1 |  |  |  |  |  |
| Age |  |  |  |  | 0.136 |
| ≤ 60 | 1 | **0.13 (0.04, 0.47)^*^** | 0.45(0.18,1.13) | 0.47 (0.18, 1.22) |  |
| > 60 | 1 | **0.12 (0.04, 0.37)^**^** | **0.37 (0.17, 0.81)^*^** | 0.62 (0.31,1.27) |  |
| BMI |  |  |  |  | 0.280 |
| ≤ 24 | 1 | **0.09 (0.03, 0.31)^**^** | **0.32 (0.14, 0.72)^*^** | **0.41 (0.19,0.88)^*^** |  |
| >24 | 1 | **0.17 (0.05, 0.55)^*^** | 0.48 (0.20, 1.16) | 0.74 (0.33, 1.65) |  |
| Smoking |  |  |  |  | 0.374 |
| Yes | 1 | **0.11 (0.04, 0.32)^**^** | **0.18 (0.07, 0.47)^**^** | 0.51 (0.25, 1.05) |  |
| No | 1 | **0.21 (0.06, 0.70)^*^** | 0.71 (0.30, 1.67) | 0.66 (0.27,1.64) |  |
| Drinking |  |  |  |  | 0.922 |
| Yes | 1 | **0.14 (0.05, 0.35)^**^** | **0.38 (0.19, 0.76)^*^** | 0.57 (0.31, 1.07) |  |
| No | 1 | 0.32 (0.07,1.41) | 0.31 (0.08, 1.29) | 0.52 (0.15,1.81) |  |
| ApoB |  |  |  |  |  |
| Age |  |  |  |  | 0.071 |
| ≤ 60 | 1 | 1.26 (0.42, 3.80) | 1.37 (0.47, 3.99) | 1.35 (0.44, 4.10) |  |
| > 60 | 1 | **4.60 (1.26,16.74)^*^** | **7.31 (2.08, 25.72)^*^** | **9.18 (2.64, 32.01)^**^** |  |
| BMI |  |  |  |  | 0.091 |
| ≤ 24 | 1 | 2.90 (0.76, 11.05) | **7.64 (2.21, 26.49) ^**^** | **4.51 (1.23, 16.50) ^*^** |  |
| >24 | 1 | 0.96 (0.34, 2.73) | 1.37 (0.52, 3.63) | **2.32 (0.93, 5.84)** |  |

**Supplementary Table 1** The multivariable associations of ApoA1, ApoB levels and ApoB/A1 ratio with T2D prevalence by subgroups (OR, 95% CI). (Continued)

| Variables | Quartiles by Apolipoproteins | | | | *p*-interaction |
| --- | --- | --- | --- | --- | --- |
|  | Q1 | Q2 | Q3 | Q4 |  |
| ApoB |  |  |  |  |  |
| Smoking |  |  |  |  | 0.806 |
| Yes | 1 | 1.05 (0.34,3.27) | **2.89 (1.09,7.68)^*^** | 2.59 (0.96,6.96) |  |
| No | 1 | 2.48 (0.72, 8.55) | **4.35 (1.37, 13.83)^*^** | **3.62 (1.11, 11.84)^*^** |  |
| Drinking |  |  |  |  | 0.294 |
| Yes | 1 | 1.35 (0.56, 3.24) | **2.82 (1.26, 6.31)^*^** | **2.20 (0.96, 5.04)** |  |
| No | 1 | 0.69 (0.09, 5.58) | 2.50 (0.47, 13.34) | 3.69 (0.70, 19.36) |  |
| ApoB/A1 ratio |  |  |  |  |  |
| Age |  |  |  |  | 0.360 |
| ≤ 60 | 1 | 0.58 (0.18, 1.89) | 1.11 (0.38, 3.19) | 1.43 (0.48, 4.21) |  |
| > 60 | 1 | 1.41 (0.64, 3.09) | 0.46 (0.17, 1.27) | **2.36 (1.08, 5.17)^*^** |  |
| BMI |  |  |  |  | 0.295 |
| ≤ 24 | 1 | 1.30 (0.51, 3.31) | 0.95 (0.36, 2.55) | **2.76 (1.14, 6.66)^*^** |  |
| >24 | 1 | 1.61 (0.63, 4.13) | 1.08 (0.40, 2.92) | 1.71 (0.65, 4.49) |  |
| Smoking |  |  |  |  | 0.878 |
| Yes | 1 | 0.89 (0.37, 2.17) | 0.41 (0.15,1.18) | 1.80 (0.78, 4.20) |  |
| No | 1 | **3.61 (1.24, 10.45)^*^** | 0.80 (0.22,2.96) | **4.10 (1.34, 12.01)^*^** |  |
| Drinking |  |  |  |  | 0.709 |
| Yes | 1 | 1.22 (0.59, 2.55) | 0.59 (0.26, 1.36) | 1.71 (0.81, 3.59) |  |
| No | 1 | 2.97 (0.66, 13.37) | 1.08 (0.20,5.93) | 3.49 (0.81, 15.11) |  |

Apo, Apolipoprotein; OR, Odds ratio; 95%CI, 95 % confidence interval; Q, quartile; T2D, type 2 diabetes. Age, BMI, education,

marriage, exercise, cigarette smoking, alcohol consumption, hypoglycemic drugs and lipid-lowering drugs use were adjusted.

^*^*p* < 0.05 compared with Q1.^**^*p* < 0.01 compared with Q1.

**Supplementary Table 2** The associations of TC, TG, HDL and LDL levels with T2D prevalence (OR, 95% CI).

| Variables | Quartiles by Apolipoproteins | | | | *p*-trend |
| --- | --- | --- | --- | --- | --- |
|  | Q1 | Q2 | Q3 | Q4 |  |
| TC |  |  |  |  |  |
| Model 1 | 1 | 1.01 (0.57, 1.81) | 1.59 (0.93, 2.72) | **1.82 (1.07, 3.08)^*^** | **0.008** |
| Model 2 | 1 | 1.19 (0.61, 2.31) | 1.46 (0.78, 2.74) | 1.54 (0.81, 2.90) | 0.145 |
| Model 3 | 1 | 1.19 (0.61, 2.30) | 1.40 (0.74, 2.64) | 1.45 (0.76, 2.75) | 0.222 |
| TG |  |  |  |  |  |
| Model 1 | 1 | 1.10 (0.62, 1.97) | 1.07 (0.60, 1.92) | **2.70 (1.61, 4.52)^**^** | **<0.001** |
| Model 2 | 1 | 0.93 (0.46, 1.90) | 1.23 (0.62, 2.41) | **2.48 (1.34, 4.60)^*^** | **<0.001** |
| Model 3 | 1 | 0.91 (0.45, 1.86) | 1.20 (0.61, 2.37) | **2.36 (1.26, 4.42)^*^** | **0.003** |
| HDL-C |  |  |  |  |  |
| Model 1 | 1 | 0.76 (0.48, 1.22) | **0.54 (0.32, 0.89)^*^** | **0.41 (0.24, 0.70)^**^** | **<0.001** |
| Model 2 | 1 | 0.63 (0.36, 1.11) | **0.48 (0.26, 0.87)^*^** | **0.31 (0.16, 0.61)^**^** | **<0.001** |
| Model 3 | 1 | 0.64 (0.37, 1.11) | **0.48 (0.26, 0.87)^*^** | **0.32 (0.16, 0.62)^**^** | **<0.001** |
| LDL-C |  |  |  |  |  |
| Model 1 | 1 | 1.43 (0.81, 2.50) | **2.32 (1.36, 3.96)^*^** | 1.29 (0.73, 2.30) | 0.172 |
| Model 2 | 1 | 1.09 (0.57, 2.09) | **1.97 (1.07, 3.61)^*^** | 1.03 (0.53, 2.00) | 0.483 |
| Model 3 | 1 | 1.07 (0.56, 2.06) | **1.88 (1.02, 3.47)^*^** | 0.98 (0.51, 1.91) | 0.611 |

TC, total cholesterol; TG, triglyceride; HDL-C, high-density lipoprotein cholesterol; LDL-C, low-density lipoprotein cholesterol;

OR, Odds ratio; 95%CI, 95 % confidence interval; Q, quartile; T2D, type 2 diabetes. Model 1 was adjusted for age. Model 2 was

adjusted for sex, age, BMI, education, marriage, exercise, cigarette smoking, alcohol consumption and hypoglycemic drugs use.

Model 3 further adjusted for lipid-lowering drugs use. ^*^*p* < 0.05 compared with Q1. ^**^*p* < 0.001 compared with Q1.
